# Influence of Film Thickness on the Structure and Properties of Copper Thin Films Deposited on BaTiO_3_ Ceramics by DCMS and HiPIMS

**DOI:** 10.3390/ma18235333

**Published:** 2025-11-26

**Authors:** Yuanhao Liao, Heda Bai, Fengtian Shi, Jin Li, Xiangli Liu

**Affiliations:** 1 School of Materials Science and Engineering, Harbin Institute of Technology (Shenzhen), Shenzhen 518055, China; 23s155037@stu.hit.edu.cn (Y.L.); 20b955008@stu.hit.edu.cn (H.B.); 23s155036@stu.hit.edu.cn (F.S.); 2 Institute of Special Environments Physical Sciences, Harbin Institute of Technology (Shenzhen), Shenzhen 518055, China; jinli2019@hit.edu.cn

**Keywords:** DCMS, HiPIMS, BaTiO_3_ ceramics, Cu film, film thickness, residual stress

## Abstract

In this study, we investigate the role of film thickness in modulating the properties of Cu films deposited on BaTiO_3_ ceramic substrates using direct current magnetron sputtering (DCMS) and high-power pulsed magnetron sputtering (HiPIMS). While HiPIMS is known for producing dense films, and the thickness-dependent properties of sputtered Cu films are well-documented, this work uniquely explores the synergistic interplay between deposition technique and thickness on BaTiO_3_ ceramic substrates, revealing novel insights into stress evolution and property optimization for advanced microelectronic and coating applications. Cu films of 300 nm, 1000 nm, and 1700 nm were systematically compared for their microstructures, surface morphologies, and electrical and mechanical properties, elucidating the critical role of thickness in densification, stress state, and overall performance. The results indicate that the target current and voltage waveforms of HiPIMS are similar to square waves, and the ionization rate is significantly higher than that of DCMS. Still, the deposition rate at the same power of 180 W is only 44.6% of that of DCMS. The films obtained by both processes present a strong (111) orientation; the crystallite size of the DCMS film grows with increasing thickness, while the HiPIMS film shows increasing and then decreasing, and its residual stress is overall lower than that of DCMS. In terms of surface morphology, DCMS films appeared porous and rough, whereas HiPIMS films were denser and smoother. In terms of properties, the resistivity of HiPIMS films is significantly lower than that of DCMS, especially at 1000 nm thickness. The binding force is also better than that of DCMS, especially at thicknesses less than 1000 nm, which is mainly attributed to the compressive stresses introduced by the energetic ion bombardment at the early deposition stage. These findings provide new mechanistic insights into thickness-dependent stress and property modulation, offering a reference for tailoring high-performance Cu films through process optimization.

## 1. Introduction

The deposition of metal films on ceramic surfaces is one of the main methods of surface metallization of mechanical alloys (SMAT) [1,2]. The deposition of a metal film on the ceramic surface enables effective bonding between ceramics and metals, thereby producing composite materials that integrate the outstanding mechanical properties of ceramics with the excellent electrical and thermal conductivity of metals [3,4]. Copper has the advantages of low resistivity, high mobility, and low cost, making it an ideal thin-film material for the metallization of ceramics [5,6,7], while BaTiO_3_ ceramics are widely used in the electronics industry due to their excellent dielectric and piezoelectric properties and thermal stability. Therefore, studying Cu films on BaTiO_3_ substrates is critical for advancing high-performance microelectronic devices and functional coatings, where precise control of film properties is essential [8].

Existing metallization processes for ceramics include silver burning [9], chemical plating [10], electroplating [11], and magnetron sputtering [12], among which magnetron sputtering has become the focus of research due to the advantages of high film quality, good bonding strength to the substrate, and process controllability [13,14,15,16]. High-power pulsed magnetron sputtering (HiPIMS), an advanced ionization physical vapor deposition technique derived from direct current magnetron sputtering (DCMS), leverages high peak power and low duty cycles to generate highly ionized plasma, enhancing film densification and structural modulation compared to DCMS [17,18,19,20,21,22]. While HiPIMS is recognized for producing dense, high-quality films, its application to Cu films on BaTiO_3_ and Si(100) substrates, particularly for thickness-dependent property evolution, remains underexplored [20,21,22].

Film thickness is an important factor affecting the microstructure and comprehensive performance of the film. While prior studies have extensively investigated the effects of power and vacuum parameters on film properties, systematic analyses of thickness-dependent effects, particularly in comparing DCMS and HiPIMS, are limited [23,24,25]. The thickness of the films is inherently influenced by the deposition process parameters such as current, power, duration, pulse number, and atmosphere. Existing research often overlooks the synergistic interplay among deposition technique, process parameters, and thickness, particularly how these factors govern stress evolution and property optimization across diverse substrates like BaTiO_3_ [26,27,28]. Moreover, most previous Cu/ceramic studies focused on single-deposition methods or single-thickness films, leaving a lack of a comprehensive understanding of how Cu films evolve structurally and functionally across thickness scales when deposited on rough ceramic substrates.

In this study, Cu films of varying thicknesses were deposited on BaTiO_3_ ceramic and Si(100) substrates by DCMS and HiPIMS. Their discharge characteristics, microstructures, surface morphologies, and electrical and mechanical properties were systematically compared. This study uniquely investigates the thickness-dependent stress evolution and property modulation under different deposition techniques. While the mechanisms of densification and metallization are well established, the current study provides insights into the effects of film thickness and deposition technique on these processes, which have not been fully explored in the context of Cu films on BaTiO_3_ and Si substrates. By addressing these gaps, the study offers a foundation for optimizing high-performance Cu/ceramic composites for microelectronics and functional coatings.

## 2. Materials and Methods

### 2.1. Sample Preparation

**Figure 1:**
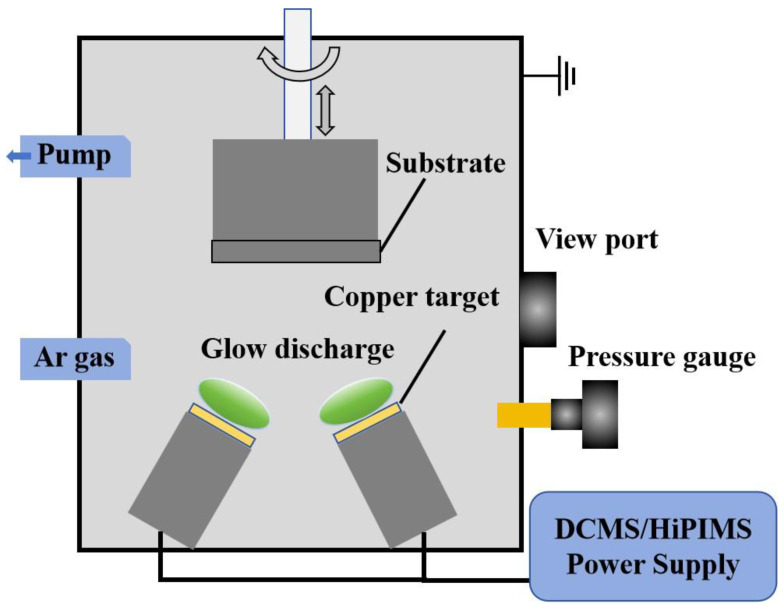
The structural diagram of the magnetron sputtering instrument.

The substrate materials were BaTiO_3_ ceramic wafers (Φ15 mm × 1.5 mm) and Si (100) wafers (10 mm × 10 mm × 0.4 mm), supplied by Kijin New Materials (Quanzhou, China). A high-purity copper target (50.3 mm in diameter, 3 mm in thickness, 99.999% purity) was obtained from Jingmai Zhongke New Material Company (Langfang, China). Thin-film deposition was performed using a magnetron sputtering equipment (DTS-200 model) manufactured by Pengcheng Semiconductor (Shenzhen, China), the structure of which is shown in Figure 1. The vacuum system consisted of a mechanical pump (8 L/s, Ningbo Baoshi Energy Equipment Co., Ltd., Ningbo, China) and a molecular pump (600 L/s, Beijing Zhongke Kaiyi Technology Co., Ltd., Beijing, China). The Ar gas (purity 99.99%) used for sputtering was supplied by Shenzhen Shente Gas Co. (Shenzhen, China).

Before deposition, the wafers were ultrasonically cleaned with anhydrous ethanol for 15 min, dried, and then placed into the deposition chamber. The chamber was evacuated for approximately 40 min until the base pressure reached 3 × 10^−3^ Pa, after which Ar gas (100 sccm) was introduced to stabilize the working pressure at 0.6 Pa. After the air pressure stabilized, the DCMS power supply was turned on to perform pre-sputtering for 5 min in order to remove impurities from the target surface. The Cu film samples with thicknesses of 300 nm, 1000 nm, and 1700 nm (samples 1–6) were deposited by DCMS and HiPIMS according to the set process parameters, and the specific parameters are shown in Table 1.

### 2.2. Characterization

During the deposition process, a digital oscilloscope (TBS100X, Tektronix, Shanghai, China) was connected to the DCMS and HiPIMS supplies to collect voltage and current signals for real-time monitoring of the target discharge characteristics. A four-channel plasma emission spectrometer (AvaSpec, Avantes, Eindhoven, The Netherlands) with a wavelength range of 200–1000 nm was used to analyze plasma species and intensities in both DCMS and HiPIMS techniques, with the probe facing the target through a high transmission film viewing window. The film thickness was measured by a step meter (Bruker-DektakXT, Bruker, Billerica, MA, USA). For cross-sectional observation in the scanning electron microscope (Gemini-SEM560, Carl Zeiss, Oberkochen, Germany), samples were prepared by first cleaving the coated substrates to expose a clean cross-sectional edge. The substrates were scored using a diamond scribe and carefully fractured to minimize mechanical damage to the film. The cleaved samples were then mounted vertically on a conductive SEM stub using carbon tape to ensure stability and electrical grounding. The surface and cross-section morphology of the films were observed using the Gemini-SEM560, and the three-dimensional morphology and roughness of the surfaces were measured using an atomic force microscope (AFM, Bruker-ICON, Bruker, Billerica, MA, USA) in Tapping Mode over multiple 10 μm × 10 μm areas, and the arithmetic mean values were taken. Crystal structures and residual stresses were analyzed by X-ray diffractometer (Rigaku-SmartLab, Rigaku Corporation, Tokyo, Japan) in grazing incidence mode (GI-XRD). Cu Kα radiation (λ = 1.5406 Å) was used, with a scanning range of 40–80°, a step size of 0.01°, a scanning rate of 4°/min, and an incidence slit of 0.5 mm. GI-XRD was selected to enhance the Cu film signal while minimizing BaTiO_3_ substrate interference [29]. Residual stresses were measured using the sin^2^ψ method, analyzing diffraction peak shifts at multiple tilt angles (ψ = 0–60°) and converting strains to stresses using Cu elastic constants (Young’s modulus: 110 GPa, Poisson’s ratio: 0.34) [30]. The Rigaku-SmartLab’s high-resolution optics system ensure accurate peak detection and thus provides reliable residual stress analysis.

The electrical properties were measured using a four-probe tester (RTS-9, Four Probes Tech, Guangzhou, China) to determine the square resistance of the films and calculate the resistivity in conjunction with the thickness, which was taken as the average value of multiple diagonal measurements. Film-substrate adhesion strength was evaluated using a multifunctional surface property tester (MFT-4000, Huahui Instruments, Lanzhou, China). The tests were performed using a conical or spherical diamond indenter to scratch the film surface at a constant speed, with the load increasing linearly with displacement, and the critical load (Lc) at which the film was peeled from the substrate was recorded. All scratch tests were performed under identical conditions: maximum load of 30 N, loading rate of 10 N/min, and scratch length of 5 mm. Each sample underwent two preliminary tests before formal measurement to eliminate potential errors caused by insufficient contact between the sample and the test base. Following this, a minimum of three valid scratch tests were conducted, with the most representative set of data selected for analysis in the paper.

Since the surface roughness of BaTiO_3_ ceramics is usually at the micron level (Ra ≈ 5 nm or higher) due to the influence of grain boundaries, domain structure, and sintering effects, this intrinsic non-uniformity is not conducive to an accurate assessment of the true surface morphology of the deposited films, in contrast to the atomic-level flatness of the Si(100) substrate surface (Ra < 0.2 nm), which provides a reliable comparative benchmark to characterize the film surface topography and roughness [31]. To more systematically and accurately compare the evolution of Cu film morphology with varying thickness and deposition methods (DCMS and HiPIMS), both surface and cross-sectional SEM samples were prepared on Si(100) and BaTiO_3_ substrates. It should be noted that only the scanning electron microscopy (SEM) characterization involved samples deposited on both Si(100) and BaTiO_3_ substrates.

## 3. Results and Discussion

### 3.1. DCMS and HiPIMS Deposited Copper Film Features

**Figure 2:**
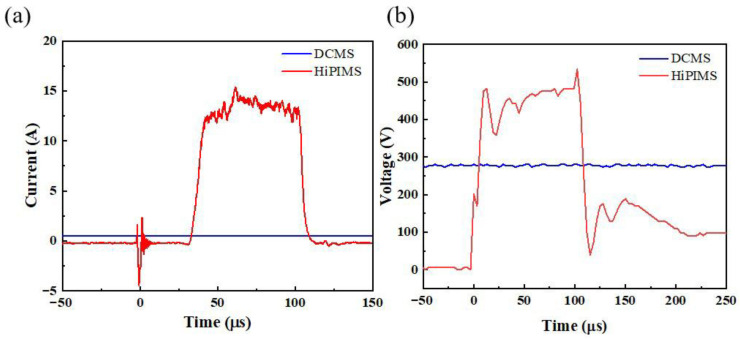
(**a**) Target current waveform diagram; (**b**) target voltage waveform diagram.

Under the same sputtering power, the target current, voltage waveforms, and deposition rates of DCMS and HiPIMS are shown in Figure 2a,b. The target current is mainly determined by the positive ions (Ar^+^, Cu^+^) and secondary electrons bombarding the target. During sputtering, the target cathode voltage attracts positive ions that bombard the target surface, generating a collision cascade within the target material and sputtering surface atoms, accompanied by secondary electron emission [32]. The escaping atoms migrate to the substrate and undergo nucleation and growth to form a thin film. As the target current increases, both the intensity of positive-ion bombardment and the amount of secondary electron emission increase, implying higher sputtering rates. As shown in Figure 2, the current and voltage waveforms of HiPIMS are close to square waves, while the waveforms of DCMS are essentially smooth. The key to this difference is the strong self-sputtering effect of HiPIMS [33]. The following conditions need to be satisfied for self-sputtering to occur:

(1) $$\prod_{\mathrm{SS}}=\alpha \beta Y_{\mathrm{SS}}=1$$

where α is the target material atom ionization rate, *β* corresponds to the ratio of the absorbed target material ions to the total target material ions; *Y_SS_* is the sputtering yield of metal ions on the target material. When the self-sputtering yield is greater than 1, a single metal-ion bombardment can trigger the generation of additional metal ions, which are returned to the target surface, forming a cyclic sputtering process. This mechanism results in a significantly higher proportion of metal ions in the HiPIMS plasma than in the DCMS, but the current and voltage waveforms show significant differences due to the pulsed discharge mode.

**Figure 3:**
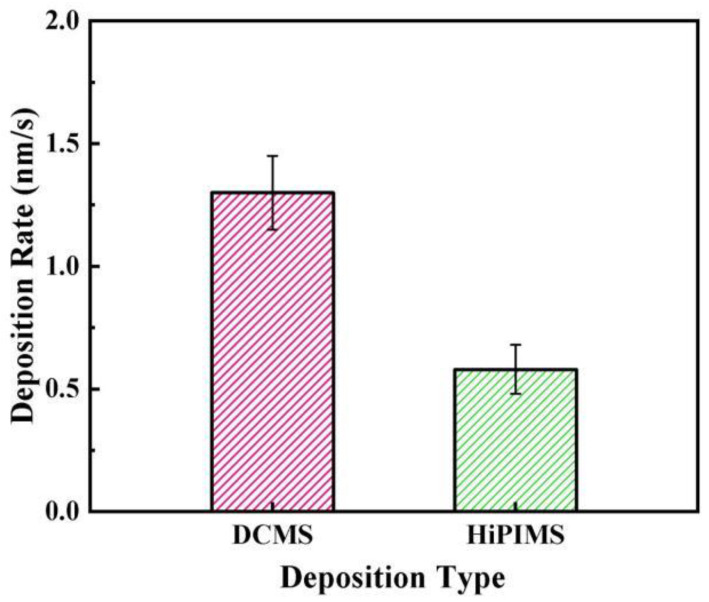
Comparison of deposition rates of DCMS and HiPIMS.

Figure 3 compares the deposition rates of the two processes. At the beginning of this study, both DCMS and HiPIMS were operated at the same power of 180 W to examine their inherent process characteristics. Under these conditions, the deposition rate of DCMS was 1.3 nm/s, while that of HiPIMS was 0.58 nm/s, accounting for only 44.6% of the DCMS rate. Although HiPIMS produces much higher instantaneous current and power densities, its pulse duty cycle is only about 3%, leading to a lower average power. In addition, the strong self-sputtering effect in HiPIMS further reduces the effective deposition rate, resulting in a lower overall efficiency than DCMS. Since the deposition rate can influence the film’s microstructure, surface morphology, and properties, additional experiments were carried out under matched deposition rates [34]. The power of DCMS was adjusted to 143 W so that its deposition rate was the same as that of HiPIMS. This adjustment made it possible to compare the two processes under equivalent growth conditions, ensuring that the following analyses reflect differences in plasma characteristics rather than in deposition rate.

**Figure 4:**
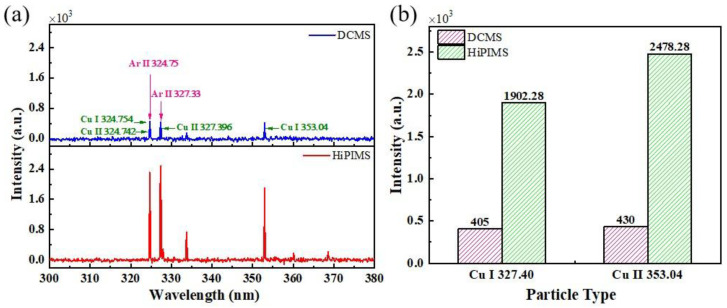
(**a**) Four-channel plasma emission spectra under DCMS and HiPIMS; (**b**) Cu I 327.40 and Cu II 353.04 relative intensity of DCMS and HiPIMS.

In order to obtain plasma information during the HiPIMS and DCMS discharge process, especially the emission characteristics of copper ions, a four-channel spectrometer was used to record plasma emission spectra. To visually compare the differences of copper atoms and ions in different power modes, the copper-related emission spectra in the range of 300–380 nm are plotted in Figure 4a. The results show that the main emission species in the plasma are Ar II, Cu I, and Cu II. Compared with DCMS, the emission intensities of Cu II, Cu I, and Ar II are significantly enhanced in HiPIMS, indicating a higher plasma density, which is attributed to the higher instantaneous power density under pulsed discharge. Further comparing the characteristic spectral lines Cu I 327.40 and Cu II 353.04 (Figure 4b), their intensities in DCMS are only 21.29% and 17.35% of those in HiPIMS, respectively.

### 3.2. Morphology and Microstructure

**Figure 5:**
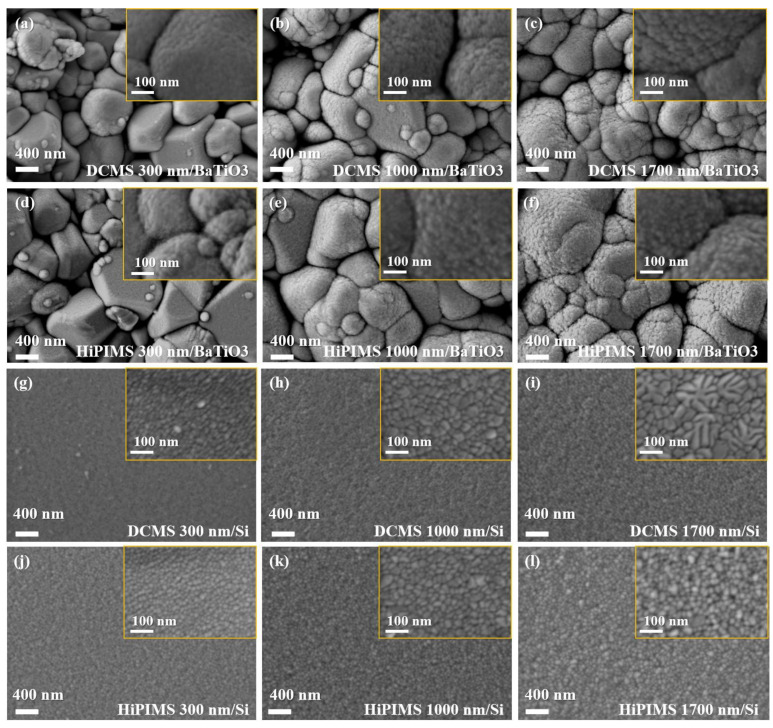
SEM surface morphology of Cu thin films deposited by DCMS and HiPIMS with different film thicknesses on two types of substrates. (**a**–**f**) Cu films on BaTiO_3_ substrates: (**a**) DCMS 300 nm; (**b**) DCMS 1000 nm; (**c**) DCMS 1700 nm; (**d**) HiPIMS 300 nm; (**e**) HiPIMS 1000 nm; (**f**) HiPIMS 1700 nm. (**g**–**l**) Cu films on Si(100) substrates: (**g**) DCMS 300 nm; (**h**) DCMS 1000 nm; (**i**) DCMS 1700 nm; (**j**) HiPIMS 300 nm; (**k**) HiPIMS 1000 nm; (**l**) HiPIMS 1700 nm.

Figure 5 shows the surface morphology of Cu films deposited on BaTiO_3_ substrates by DCMS and HiPIMS with thicknesses of 300 nm, 1000 nm, and 1700 nm, respectively. As shown in Figure 5a–f, when the film thickness is relatively small, the surface morphology of the Cu films closely resembles the original granular texture of the BaTiO_3_ substrate. With increasing deposition time and film thickness, obvious traces of film coverage appear on the BaTiO_3_ grains, and the Cu particle size gradually increases. This morphological evolution can be attributed mainly to the increased deposition time and the gradual rise in substrate temperature during sputtering. A longer deposition time allows the Cu nuclei to continuously grow and extend across the BaTiO_3_ grains, while the elevated temperature promotes surface atom mobility, leading to a gradual enlargement and merging of surface particles. In addition, the increased continuity of the film surface reduces the direct influence of the underlying ceramic grains, allowing the intrinsic growth characteristics of Cu to become more apparent at higher thicknesses. As a result, Cu grains become larger, and the film surface becomes more continuous and well-covered at higher thicknesses. Compared with DCMS, the films deposited by HiPIMS show a more compact and smoother morphology, which is consistent with the higher energy of the ion flux involved in the process.

As discussed in Section 2.2, Si(100) substrates were also used in this study as atomically smooth reference surfaces to eliminate the influence of substrate roughness on morphological characterization. Figure 5g–l show the surface morphology of Cu films deposited on Si(100) substrates under the same deposition conditions. Although the focus of this work is on BaTiO_3_-based films, the inclusion of Si(100) substrates enables clearer observation of intrinsic Cu film growth behavior and the effect of deposition method (DCMS vs. HiPIMS). This complementary comparison further supports the interpretation of morphology evolution presented for the BaTiO_3_ system.

**Figure 6:**
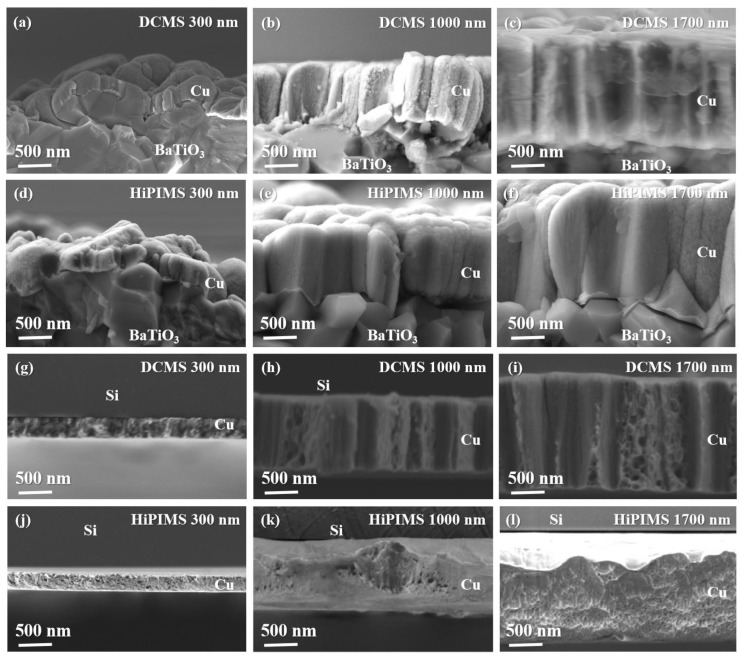
Cross-sectional SEM images of Cu thin films deposited by DCMS and HiPIMS on BaTiO_3_ and Si(100) substrates with different film thicknesses. (**a**–**f**) Cu films on BaTiO_3_ substrates: (**a**) DCMS 300 nm; (**b**) DCMS 1000 nm; (**c**) DCMS 1700 nm; (**d**) HiPIMS 300 nm; (**e**) HiPIMS 1000 nm; (**f**) HiPIMS 1700 nm. (**g**–**l**) Cu films on Si(100) substrates: (**g**) DCMS 300 nm; (**h**) DCMS 1000 nm; (**i**) DCMS 1700 nm; (**j**) HiPIMS 300 nm; (**k**) HiPIMS 1000 nm; (**l**) HiPIMS 1700 nm.

For the same deposition method, the particle size gradually increases with the increase in film thickness. This is mainly due to the longer deposition time and the substrate temperature rise induced by Ar^+^ bombardment, which promotes the particle growth. At the same thickness, the films obtained by the two deposition methods show significant differences. For example, the 300 nm DCMS film (Figure 5g) already shows coarse, unevenly fused particles. With increasing thickness up to 1700 nm, the surface island particles further merge and take on a branching morphology. This results from the high deposition rate and predominantly neutral Cu atoms in DCMS (ionization rate <10%, energy <10 eV). These atoms possess limited surface mobility, nucleating randomly and favoring three-dimensional island growth. As thickness increases, insufficient lateral diffusion leads to enhanced roughness [35,36,37]. In contrast, the surface of films deposited by HiPIMS exhibits a dense and continuous structure. This is attributed to its high ionization rate (>50%), which generates a large number of Cu ions with energies higher than 50 eV [38]. The bombardment of these energetic ions promotes the rearrangement of atoms on the surface and fills up the voids at the grain boundaries, thus effectively inhibiting island growth and promoting film densification [39]. This can also be verified from the SEM image of the film cross-section (Figure 6): the DCMS-deposited film shows a columnar and porous structure with distinct inter-particle voids (Figure 6i), indicating insufficient surface diffusion and limited densification during growth. In contrast, the HiPIMS-deposited film exhibits a continuous and compact cross-section, reflecting strong ion-assisted adatom rearrangement and the more effective filling of inter-grain gaps. The pronounced difference in film morphology highlights the influence of ionization degree and particle energy on the microstructure evolution of sputtered Cu films. It is well known that film porosity can exacerbate electromigration, electrical resistance drift, and optical property degradation, ultimately reducing device reliability. Therefore, minimizing void formation during deposition is essential for ensuring stable performance in microelectronic and functional coating applications. At the same sputtering power, the HiPIMS technique demonstrates significant advantages in improving film density and surface quality due to its high-energy ion bombardment and enhanced adatom mobility, which collectively contribute to the formation of a high-integrity Cu layer [40,41].

**Figure 7:**
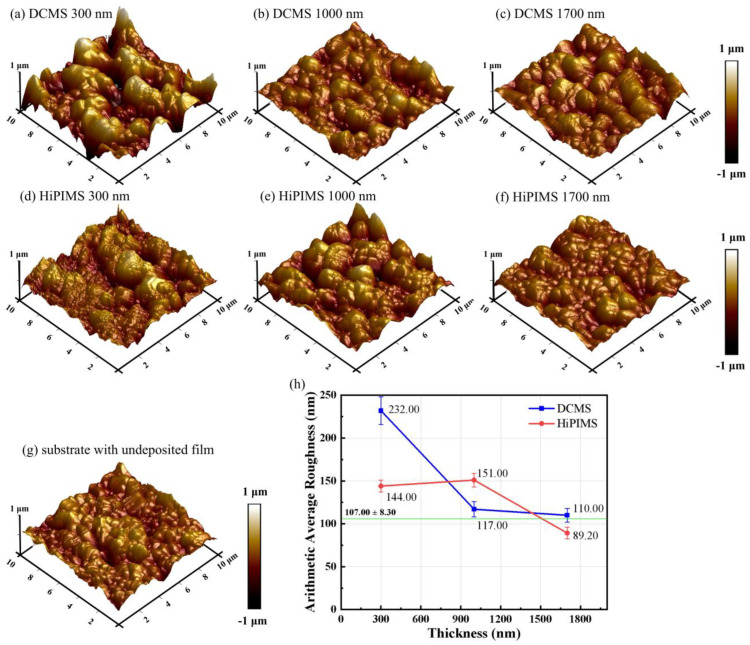
(**a**) AFM surface morphology of 300 nm films deposited by DCMS; (**b**) AFM surface morphology of 1000 nm films deposited by DCMS; (**c**) AFM surface morphology of 1700 nm films deposited by DCMS; (**d**) AFM surface morphology of 300 nm films deposited by HiPIMS; (**e**) AFM surface morphology of 1000 nm films deposited by HiPIMS; (**f**) AFM surface morphology of 1700 nm films deposited by HiPIMS; (**g**) the roughness of BaTiO3 ceramics; (**h**) the roughness of Cu films deposited by DCMS and HiPIMS. The green line in Figure (**h**) indicates the roughness of the BaTiO_3_ substrate.

The evolution of surface roughness with increasing film thickness (Figure 7) exhibits a non-monotonic trend for HiPIMS-deposited films, in contrast to the pronounced monotonic decrease observed for DCMS. This behavior is consistent with the SEM observations: DCMS films gradually smoothen as thickness increases, whereas HiPIMS films undergo a more complex surface evolution.

For DCMS, the roughness decreases from 232 nm at 300 nm to 117 nm at 1000 nm and stabilizes near 110 nm at 1700 nm, reflecting the progressive filling of substrate asperities and the gradual coalescence of columnar grains. In contrast, HiPIMS films show roughness values of 144 nm, 151 nm, and 89.2 nm at thicknesses of 300, 1000, and 1700 nm, respectively. The higher roughness at 1000 nm corresponds to the more textured morphology observed in SEM and indicates a transient roughening stage during intermediate growth. This non-monotonic behavior arises from the interplay between ion-induced re-nucleation and surface diffusion [42]. At early thicknesses, the film partially replicates the rough ceramic substrate and experiences repeated re-nucleation under pulsed high-ion-flux conditions, leading to moderate roughness [43]. At intermediate thickness, frequent re-nucleation events temporarily enhance height variations, producing the highest Ra. With further growth, energetic ion bombardment promotes adatom mobility, grain coalescence, and surface smoothing, resulting in the lowest roughness at 1700 nm [37].

**Figure 8:**
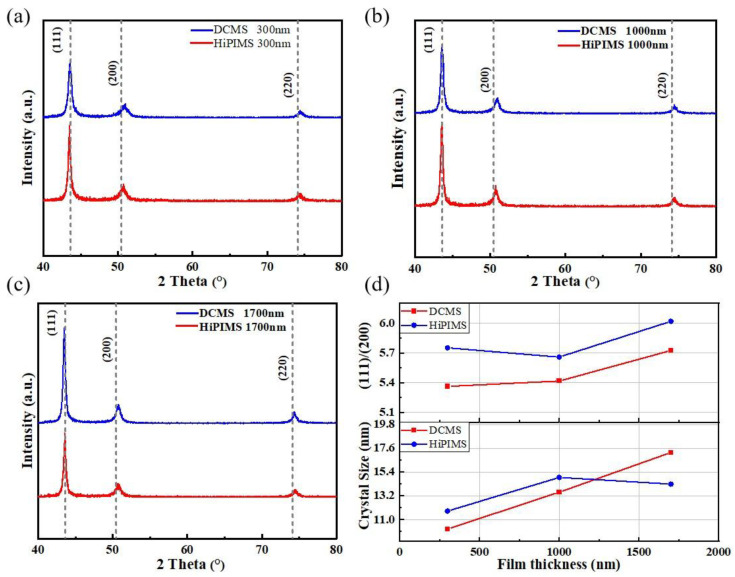
(**a**) XRD of 300 nm copper films deposited by DCMS and HiPIMS; (**b**) XRD of 1000 nm copper films deposited by DCMS and HiPIMS; (**c**) XRD of 1700 nm copper films deposited by DCMS and HiPIMS; (**d**) peak intensity ratio of Cu (111)/(200) and crystallite size.

Figure 8 shows the XRD spectra of Cu films with different thicknesses deposited on BaTiO_3_ substrates by DCMS and HiPIMS, along with the Cu(111)/(200) peak intensity ratio and crystallite size results. As can be seen from Figure 8a–c, the films exhibit a significant preferential orientation of the (111) crystal plane as the film thickness increases from 300 nm to 1700 nm, regardless of the deposition method used. This is because the (111) facet has the lowest surface energy in the face-centered cubic structure, and thus it is more likely to grow preferentially during film deposition [44].

Based on Scherrer’s formula [45], the half-height width of the diffraction peaks was calculated to estimate the crystallite size under different process and thickness conditions, and the results are shown in Figure 8d. The Scherrer formula is as follows:

(2) $$D=\frac{K\gamma }{\text{Bcos}\theta \prime }$$

where *D* is the crystallite size, *K* is the Scherrer constant, *B* is the half peak width or integrated width of the diffraction peak of the measured sample, *θ* is the Bragg angle, and *γ* is the wavelength of the X-rays.

Crystallite size, representing the size of the smallest crystalline domains within the Cu films, was determined using Scherrer’s equation, which relates the broadening of XRD peaks to the size of these domains. Overall, whether it is DCMS or HiPIMS, the crystallite size increases gradually with the increase in film thickness. However, under the HiPIMS process, the crystallite size decreases as the film thickness increases to 1700 nm. This reduction may be attributed to the accumulation of dislocations and the formation of subgranular boundaries due to long-term bombardment by energetic ions, which resulted in the refinement of large crystallites into nanocrystals [46]. This phenomenon suggests that although HiPIMS helps to form dense films, excessive ion bombardment may also trigger crystallite refinement and an increase in structural defects, thus affecting their crystalline integrity.

XRD is a highly accurate and feasible method for measuring residual stresses in Cu/BaTiO_3_ systems, offering non-destructive analysis with precision (error < ±10 MPa) [47,48]. The stress (*σ*) was calculated using the equation:

(3) $$\sigma =\frac{E}{\left(1+\nu \right)}\cdot \frac{1}{d_{0}}\cdot \frac{\partial d_{\psi }}{\partial \sin^{2}\psi }$$

where *E* is the Young’s modulus of Cu (110 GPa); *ν* is the Poisson’s ratio (0.34); *d*_0_ is the unstressed lattice spacing; and *d_Ψ_* is the lattice spacing at tilt angle *Ψ*. This formula relates peak shifts to stress via elastic constants, ensuring accurate quantification.

**Figure 9:**
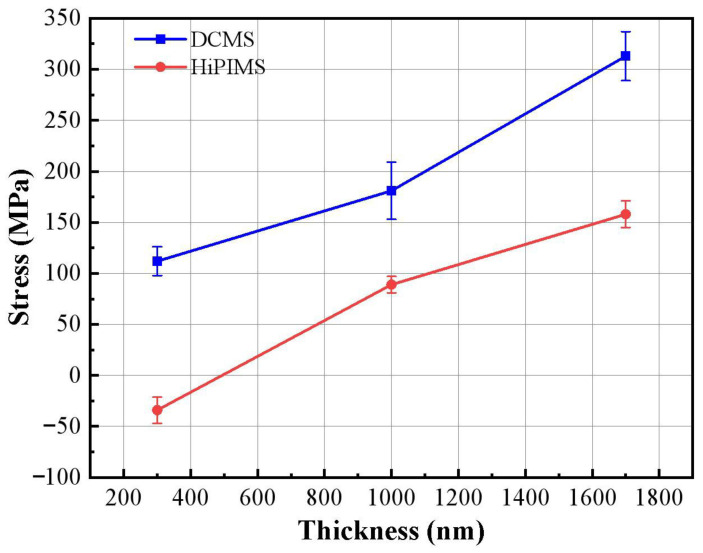
Residual stress of Cu thin films of different thicknesses deposited by DCMS and HiPIMS.

Recent studies, such as Ren et al. (2024) on Cu films on Si(100) substrates [49] and Liu et al. (2024) on SiC ceramic substrates [50], validate the reliability of *sin*^2^*ψ* for such applications. The internal stresses of Cu films were measured by XRD under different process and thickness conditions, and the results are shown in Figure 9. For DCMS films, the residual stresses remain tensile across all thicknesses, increasing from 112 ± 14 MPa at 300 nm to 181 ± 28 MPa at 1000 nm, and reaching 313 ± 24 MPa at 1700 nm. This continuous rise is mainly attributed to the thermal stress caused by the mismatch in thermal expansion coefficients between Cu and BaTiO_3_, which outweighs the relatively weak compressive stress introduced by low-energy ion bombardment. In contrast, the HiPIMS films show a clear compressive-to-tensile transition: at 300 nm, the residual stress is −34 ± 13 MPa, indicating strong compressive stress; at 1000 nm, it shifts to a moderate tensile value of 89 ± 8 MPa, and further increases to 158 ± 12 MPa at 1700 nm. The significant compressive stress at 300 nm is induced by the high ionization rate (>50%) of HiPIMS, which generates abundant energetic ions that bombard the growing film, producing an “atomic shot peening effect” that densifies the microstructure and introduces compressive strain [51,52]. As the film becomes thicker, the ion-induced compressive contribution weakens, while tensile stress arising from thermal expansion mismatch accumulates, ultimately causing the overall stress state to transition from compressive to tensile.

These results indicate that HiPIMS can effectively regulate the stress state during the early growth stage, producing compressive stress in thin layers that promotes densification and enhances interfacial bonding. However, the diminishing compressive effect at larger thicknesses suggests that additional process optimization may be necessary for maintaining favorable stress conditions in thicker films.

### 3.3. Performance

**Figure 10:**
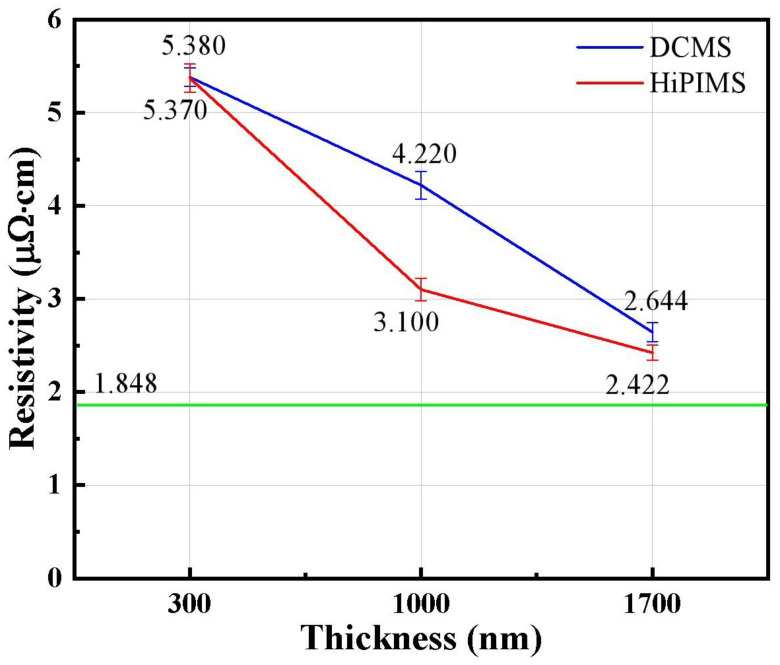
Resistivity of Cu thin films of different thicknesses deposited by DCMS and HiPIMS. The green line indicates the intrinsic resistivity of the Cu film.

Figure 10 shows the resistivity of Cu films of different thicknesses deposited by DCMS and HiPIMS. The intrinsic resistivity of copper thin film is 1.848 μΩ·cm, as indicated in Figure 10. It can be seen that the resistivity decreases with the increase in film thickness in both processes. At 300 nm thickness, the resistivities of DCMS and HiPIMS films were measured as 5.380 μΩ·cm and 5.370 μΩ·cm, respectively; when the thickness increased to 1700 nm, the resistivities decreased by 50.86% and 54.90%, respectively. Although both values remain higher than the bulk Cu resistivity (1.673–1.848 μΩ·cm), the decreasing trend reflects the gradual reduction in electron scattering as the films become thicker and more continuous. The stress evolution in Figure 9 correlates well with these changes. DCMS films maintain relatively high tensile stress (112–313 MPa), while HiPIMS films shift from compressive (−34 MPa) to moderate tensile stress (158 MPa). The lower stress state and higher densification efficiency of HiPIMS contribute to its consistently lower resistivity—for instance, 3.100 μΩ·cm at 1000 nm, compared with 4.220 μΩ·cm for DCMS.

The resistivity behavior can be quantitatively explained using the Mayadas–Shatzkes (M–S) grain-boundary scattering model [53], which describes how resistivity increases with grain-boundary density and electron-reflection coefficient (R). At small thicknesses, the Cu films contain smaller grains and more grain boundaries, leading to strong scattering. As the thickness increases, grain coalescence reduces boundary density, lowering resistivity for both deposition methods.

HiPIMS provides additional mechanisms for conductivity improvement. The high ionization rate and energetic ion bombardment inherent to HiPIMS enhance adatom mobility, promote lateral grain growth, suppress boundary voids, and smooth the surface [37,54], all of which reduce the effective R parameter in the M–S model. As a result, HiPIMS films achieve superior electrical performance compared with DCMS at identical thicknesses, making them promising for interconnects and high-frequency device applications.

**Figure 11:**
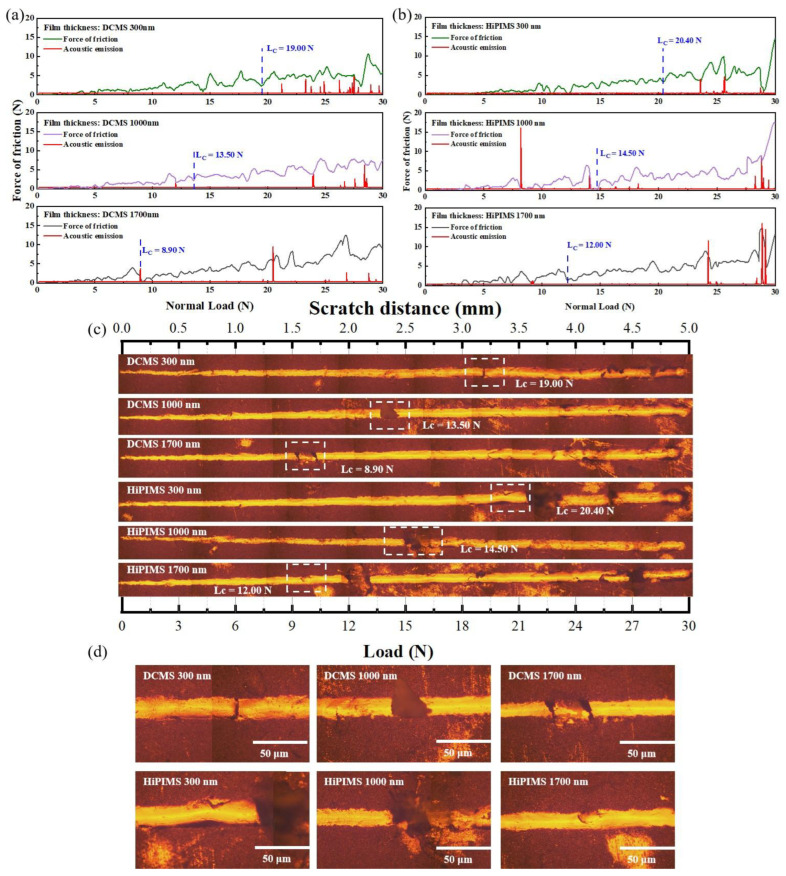
(**a**) Typical loading-friction curves of 300 nm, 1000 nm, and 1700 nm copper films prepared by DCMS; (**b**) typical loading-friction curves of 300 nm, 1000 nm, and 1700 nm copper films prepared by HiPIMS; (**c**) scratch morphology of copper films with different thicknesses deposited by DCMS and HiPIMS; (**d**) detailed scratch morphology of copper films with different thicknesses deposited by DCMS and HiPIMS.

The scratch test is a commonly used semi-quantitative technique for evaluating the bonding strength at the film–substrate interface [55]. It is based on the observation of scratch morphology by optical microscopy and the determination of the critical load (Lc) that induces film failure, in conjunction with friction-load force curves, to assess bonding strength [56,57,58]. Figure 11 presents the friction-load force curves and scratch morphologies of Cu films with thicknesses of 300 nm, 1000 nm, and 1700 nm deposited by DCMS and HiPIMS.

The results show that the bonding strength of Cu films deposited by both processes decreases with increasing thickness, while HiPIMS films exhibit the highest bonding strength at 300 nm. This behavior is primarily attributed to the energetic ion bombardment associated with HiPIMS, which enhances interfacial densification and promotes strong mechanical interlocking and chemical bonding with the substrate. At this early deposition stage, the HiPIMS film also exhibits a slight compressive residual stress (−34 MPa, Figure 9), which contributes to stabilizing the film–substrate interface and corresponds to the highest measured Lc (20.40 N). In contrast, the DCMS film at 300 nm already possesses a significant tensile stress (112 MPa), consistent with its lower Lc value (19.00 N). As thickness increases from 300 nm to 1000 nm, the residual stress in HiPIMS films shifts from compressive to moderate tensile stress (89 MPa), and the Lc correspondingly decreases to 14.50 N. A similar trend is observed for DCMS: its tensile stress rises to 181 MPa at 1000 nm, accompanied by a reduction in Lc to 13.50 N. These results demonstrate that the evolution toward higher tensile stress in both films weakens interfacial stability, which is reflected quantitatively in the decreasing bonding strength. It should also be noted that tensile-stress accumulation is often associated with grain boundary voids and microcrack formation, which further accelerates interfacial weakening under increasing thickness. For thicker films (1700 nm), the DCMS film exhibits the highest tensile stress (313 MPa), and its Lc decreases sharply to 8.90 N, indicating severe interfacial degradation driven by tensile-stress concentration. Meanwhile, the HiPIMS film reaches a tensile stress of 158 MPa and an Lc of 12.00 N, showing that its relatively lower tensile stress corresponds to better adhesion compared with DCMS at the same thickness. The transition from compressive to tensile stress in HiPIMS films, combined with densification effects that diminish with thickness, explains the gradual decline in adhesion performance.

In summary, the quantitative correspondence between residual stress and Lc demonstrates that compressive or low-magnitude tensile stress contributes to stronger adhesion, whereas high tensile stress promotes interfacial failure. HiPIMS shows clear advantages in controlling stress evolution and maintaining strong adhesion, especially for Cu films thinner than 1000 nm, making it promising for high-reliability interconnects and functional coating applications. Overall, these findings provide valuable guidance for optimizing deposition parameters to achieve tailored stress states and improved adhesion performance in Cu-based thin-film systems.

## 4. Conclusions

This paper systematically compared the discharge characteristics, microstructure, and overall properties of Cu films deposited by DCMS and HiPIMS at different thicknesses and revealed the mechanisms by which deposition method and thickness jointly regulated film densification, stress state, and overall performance. Unlike previous Cu/ceramic studies that typically focus on a single deposition technique or single-thickness films, this work provides a multi-thickness, dual-technique comparison that clarifies how Cu films evolve on a rough ceramic substrate and establishes quantitative links between stress, adhesion, and resistivity. The results show the following:

(1) HiPIMS films exhibit square waveform target current and voltage characteristics during the deposition process, and the ionization rate is significantly higher than that of DCMS, although the deposition rate was lower.
(2) The Cu films deposited by both processes presented (111) texture, and the HiPIMS films showed compressive stress at 300 nm, which gradually changes to tensile stress with the increase in thickness, while the DCMS film is always tensile, indicating that HiPIMS is more suitable for thin-layer stress modulation.
(3) Compared with DCMS films, HiPIMS films possessed finer and denser grains, and showed reduced resistivity, with the most pronounced differences observed at a thickness of 1000 nm.
(4) The film bonding decreased with thickness, but HiPIMS showed better bonding in the range of 300–1000 nm, which is mainly due to the compressive stress introduced by ion bombardment in the early deposition stage.

Overall, this study clarifies the synergistic interplay between deposition method and film thickness in regulating stress evolution and performance of Cu films on ceramic substrates. The demonstrated ability of HiPIMS to achieve dense, low-resistivity, and well-adhered Cu films at sub-micron to micron-scale thicknesses highlights its strong potential for next-generation microelectronic metallization and functional ceramic coatings, thereby strengthening the conceptual and application-level significance of this work.

## Figures and Tables

**Table 1:** The sample parameters.

	Power Mode	Pressure (Pa)	Average Power (W)	Duty Cycle	Thickness (nm)	Temperature (°C)
1	DCMS	0.6	143	100%	300	23
2					1000	
3					1700	
4	HiPIMS		180	3%	300	
5					1000	
6					1700	

## Data Availability

The original contributions presented in this study are included in the article. Further inquiries can be directed to the corresponding author.
